# Women’s Autonomy and Its Correlates in Western Nepal: A Demographic Study

**DOI:** 10.1371/journal.pone.0147473

**Published:** 2016-01-22

**Authors:** Tulsi Ram Bhandari, V. Raman Kutty, T. K. Sundari Ravindran

**Affiliations:** 1 Department of Public Health, School of Health and Allied Sciences, Pokhara University, Lekhnath-12, Kaski, Nepal; 2 Achutha Menon Centre for Health Science Studies, Sree Chitra Tirunal Institute for Medical Sciences and Technology, Trivandrum, Thiruvananthapuram – 695011, Kerala, India; University of Westminster, UNITED KINGDOM

## Abstract

Despite various efforts for enhancing women’s autonomy in developing countries, many women are deprived of their capacity in decision-making on their household affairs as well as social issues. This paper aimed to examine women’s autonomy and its associated factors in the Kapilvastu district of Nepal. We measured women’s autonomy using a recently developed women’s autonomy measurement scale from June to October 2014. Descriptive statistics, chi-square test and logistic multivariate modeling technique were applied for assessing the association of demographic and socio-economic characteristics of women and their autonomy. Mean score for women’s autonomy was 23.34± 8.06 out of the possible maximum 48. It was found to be positively associated with higher age difference at marriage, advantaged caste/ethnicity, better employment for the husband, couple’s education more than 10 years schooling, and higher economic status of the household. We found strong direct effect of women’s education (OR = 8.14, CI = 3.77–17.57), husband’s education (OR = 2.63, CI = 1.69–4.10) and economic status of household (OR = 1.42, CI = 1.01–2.03) on women’s autonomy. When we adjusted women’s education for husband’s education, the odds ratio decreased by around 22% {from (OR = 8.14, CI = 3.77–17.57) to (OR = 6.32, CI = 2.77–14.46)} and was a mediator effect. The economic status of household also had mediator effect on women’s autonomy through their education. Education status of women is a key predictor of women’s autonomy in Kapilvastu district. Husband’s education and economic status of the household are other important predictors of women’s autonomy which have a mediator effect on women’s autonomy. Improving educational status and economic conditions of both women and their husbands may be the best solution to promote women’s autonomy.

## Introduction

Autonomy is a multidimensional concept and difficult to quantify[1]. It refers to the independence or freedom of will of one’s action. It is also the ability of a person to act independently in accordance with objective morality rather than under the influence of desires[2]. It is defined as technical, social, and psychological ability for making decisions about one’s private concerns as well as that of one’s intimates[3].

Women’s autonomy is a broad as well as complex term which has a contextual meaning. It is influenced by personal attributes of women as well as socio-cultural norms and values of the society[4]. Prior literature focused on education, occupation and demographic characteristics i.e. age at marriage, age difference at marriage, numbers of children, sex of children and so forth for measuring the women’s autonomy[5].

Writers, researchers and social activists had different view on the definition and measurement procedures of women’s autonomy. Most researchers preferred proxy indicators such as educational attainment, employment, income, spousal age difference and type of family to assess women’s autonomy in decision making, use of financial and physical resources and freedom of movement, and utilization of maternal health care services[6,7]. More recently, women’s autonomy has been defined as enacted ability of women to influence in decision-making, control over financial resources and freedom of movement[8–10].

Women’s better educational and occupational status and spousal support for seeking maternal health care associated positively with women’s autonomy in Nepal. In the same way, movement, decision-making and financial autonomy and autonomy for spousal communication also had positive influence on the utilization of maternal health care services[10,11].

Low autonomy status of women associated with poor access to their basic needs i.e. food, cloths, education, health and security[12,13]. In most south-east Asian countries, women had an inferior position and less autonomy than man at the household level as well as in society[10,14]. As a result, women cannot access even to their basic needs and claim their rights without prior permission of either their husbands or other senior members of family.

Women’s personal autonomy contributes to create a conducive social environment in decision making and controlling over the financial and physical resources[15]. Furthermore, it enhances access to information. Similarly, women’s empowerment programs aid to increase the decision-making power as well as negotiation capacity in making decisions at the household level and in society. Empowerment is a process, requiring change over time from one state to another[16].

Despite various efforts on enhancing the women’s autonomy, many women are restricted in making decisions on household affairs as well as on social issues, utilize financial and physical resources and freedom of movement in developing countries. Literature also shows the similar situation of women’s autonomy in Nepal. This paper aimed to examine women’s autonomy and factors associated factors with it in Kapilvastu district of Nepal.

## Materials and Methods

We conducted a population based cross-sectional survey to assess autonomy of women and associated factors in Kapilvastu district of Nepal using a newly developed women’s autonomy measurement scale by authors. This work is a part of Ph.D. research of the first author in Sree Chitra Tirunal Institute for Medical Sciences and Technology Trivandrum, Kerala, India. The Institute Ethical Committee of Sree Chitra Tirunal Institute for Medical Sciences and Technology (SCTIMST) gave ethical clearance for this study. Nepal Health Research Council (NHRC) is a local authority for ethical clearance in Nepal. NHRC and SCTIMST follow the same guideline of the Indian Council of Medical Research (ICMR) for ethical review. Hence, we took written permission from District Public Health Offices of the Rupandehi and Kapilvastu districts, the concerned local authorities of Nepal before initiating the research work. All participants were explained about the study and took written informed consent before they participated by singing/providing thumb impression on the consent form which was reviewed and approved by the Ethical Review Committee.

### Brief description of scale construction

We conducted a population based cross-sectional study in Ilaka (subdivision of district) number eleven of Rupandehi district, Nepal for construction and validation of a women’s autonomy measurement scale. The scale consists of three dimensions of women’s autonomy i.e. decision making autonomy, financial autonomy and freedom of movement autonomy. After intensive literature review and consultation with the experts, we prepared a preliminary draft of the scale which consists of 24 items (S1 Table adopted from [17]). Each item is scored zero to two (0- dependent/ always, 1- joint/sometime and 2- independent/never), so that the possible score is minimum zero to the maximum forty eight. Based on the (1: 10) ratio of items and respondents, we administered the scale to 250 married women of reproductive age. Scale development process followed various steps i.e. definition of construct, generation of items pool, pretest, psychometric tests analysis and validation. The scale’s characteristics included Cronbach’s Alpha value (0.84), average content validity ratio/ index (0.8) and overall agreement- Kappa value of the items (0.83), which were acceptable.

### Study setting, population and data collection

We executed a population based cross-sectional survey in Kapilvastu district of Western Development Region, Nepal for assessing the women’s autonomy and associated factors. This is a part of a large study on “women’s autonomy and utilization of maternal health care services in Kapilvastu District of Nepal.” The target population comprised of married women of reproductive age who had full term delivery within a year and had completed their postnatal period. Sample size was fixed based on the proportion of skilled care at birth (15.92%) of Kapilvastu district, [18] with design effect = 2 and non-response rate = 20% using online OpenEpi statistics software[19]. We selected ten village development committees (VDCs), out of 76 VDCs of the district using simple random sampling (lottery) method. The final number of women at VDC level was fixed proportionately based on the population of VDC. We followed convenience sampling method and interviewed 500 women from 10 VDCs using the newly developed women’s autonomy measurement scale. For identifying the respondents in each village, first, we consulted the local people to identify the center of the village. We went to the center and started data collection in a randomly chosen direction. We continued the household visit in the clock-wise direction until obtaining the required number of respondents.

### Study variables and statistical analysis

The units of analysis of this study were women who delivered at least one child within a year preceding the survey. After reporting descriptive statistics, we compared sum score of women’s autonomy with the selected demographic and socio-economic characteristics of respondents. We computed mean score, standard deviation and p-value applying one-way analysis of variance (ANOVA) test to assess the association of women’s autonomy with various explanatory variables. We performed multivariate modeling to examine how the socio-economic variables influenced on the women’s autonomy. We chose women’s education, husband’s education and economic status of women as key predictor variables from couple’s education, couple’s occupation and economic status of the household to construct logistic regression models. First, we created two categories for the respondent’s education considering illiterate or less than ten years schooling as ‘less educated’ and ten or more years schooling as ‘educated’. Then, we followed same criteria for husband’s education. Similarly, we converted economic status into two categories considering median value of socio-economic status measurement score as cutoff point. We considered women below the median as ‘low economic status and above the median as ‘high economic status’. Hence, we constructed model-I between respondent’s education and their autonomy. We further adjusted women’s education for husband’s education to construct model-II, and husband’s education and economic status of the household to construct model-III respectively. After building models, we analyzed various pathways to establish the association between women’s autonomy, and women’s education, husband’s education and economic status of the household.

### Data Availability

All relevant data of the paper and supporting information are available in files S1 and S2 Data Files.

## Results

We measured women’s autonomy using a recently developed three-point Likert-type scale by authors. We further collected the demographic and socio-economic information using structured interview schedule. The mean score of women’s autonomy score was found 23.34± 8.06 out of a possible maximum of 48. We compared association of the sum score of women’s autonomy with the selected demographic and socio-economic factors using one-way ANOVA test. It can be seen that some demographic factors (Table 1) and most of socio-economic factors (Table 2) emerged as statistically significant.

**Table 1:** Demographic characteristic of women and their autonomy (n = 500).

Predictors		No. women	Per cent	Mean± SD of sum score of women’s autonomy	p value
Age in Year					0.73
	<20 year	61	12.2	21.57±7.93	
	20–35 year	399	79.8	22.44±7.94	
	>35 year	40	8	22.53±9.41	
	Total	500	100	22.34±8.05	
Age at marriage					<0.05*
	<20 year	427	85.4	22.04±7.98	
	20 year & above	73	14.6	24.12±8.33	
	Total	500	100	22.34±8.05	
Age difference at marriage					<0.001*
	5 or less	473	94.6	22.05±7.80	
	>5 year	27	5.4	27.41±10.57	
		500	100	22.34±8.05	
Age at first pregnancy					<0.05*
	<20 year	294	58.8	21.65±8.00	
	20 year & above	206	41.2	23.34±8.04	
	Total	500	100	22.34±8.05	
Number of parity					0.135
	1 to 2	239	47.8	22.93±7.90	
	3 to 4	153	30.6	22.20±8.34	
	5 or more	108	21.6	21.12±7.89	
	Total	500	100	22.34±8.05	
Birth spacing(n = 365)					<0.05*
	<24 months	139	39.04	20.68±8.71	
	24 to 36 months	116	32.58	22.90±7.05	
	>36 to 60 months	91	25.56	21.80±7.76	
	>60 months	19	5.34	26.10±8.04	
	Total	365	100	21.95±8.03	
Native language					<0.001*
	Nepali	74	14.80	28.45±7.68	
	Awadhi	401	80.20	21.02±7.60	
	Tharu	25	5.00	25.56±7.20	
	Total	500	100	22.34±8.05	
Religion					<0.05*
	Hindu	437	87.40	22.67±8.11	
	Muslim	63	12.60	20.03±7.32	
	Total	500	100	22.34±8.05	
Caste/ethnicity					<0.001*
	Dalit	19	3.80	21.74±12.80	
	Disadvantaged Janajatis (tribal population)	47	9.40	23.80±7.83	
	Disadvantaged non-Dalit Terai ethnicity	380	76.00	21.15±7.52	
	Advantaged ethnicity	54	10.80	29.67±5.54	
	Total	500	100	22.34±8.05	

* *One-way ANOVA test significant at p<0*.*05*

** *One-way ANOVA test significant at p<0*.*001*

**Table 2:** Socio-economic characteristic of women and their autonomy (n = 500).

Predictors		No. of women	Per cent	Mean± SD of sum score of women’s autonomy	p value
Education status					< .001**
	Illiterate	292	58.4	20.64±7.42	
	<5 years schooling	119	23.8	21.76±8.43	
	5–10 years schooling	70	14	28.57±6.53	
	>10 years schooling	19	3.8	29.16±5.51	
	Total	500	100	22.34±8.06	
Education status of husband					<0.001**
	Illiterate	105	21	21.13±7.89	
	<5 years schooling	190	38	20.92±7.71	
	5–10 years schooling	182	36.4	23.79±8.15	
	>10 years schooling	23	4.6	28.26±6.46	
	Total	500	100	22.34±8.06	
Occupation					<0.001**
	Agriculture	91	18.2	19.67±9.22	
	Service	9	1.8	26.22±9.72	
	Own business	19	3.8	27.74±7.53	
	Migrant worker/labourer	23	4.6	22.30±8.20	
	Housewife	358	71.6	22.64±7.50	
	Total	500	100	22.34±8.06	
Occupation of husband					<0.001**
	Agriculture	260	52	20.81±8.14	
	Service	45	9	28.67±6.22	
	Own business	61	12.2	23.59±7.92	
	Migrant worker/labourer	57	11.4	22.89±7.92	
	Overseas employee	77	15.4	22.43±7.90	
	Total			22.34±8.06	
Wealth index					<0.05*
	Poor	294	58.80	21.49±7.93	
	Medium class	184	36.80	23.18±8.21	
	Rich	22	4.40	26.68±6.26	
	Total	500	100	22.34±8.05	

* One-way ANOVA test significant at p<0.05

** *One-way ANOVA test significant at p<0*.*001*

Form multivariate analysis, odds ratios (OR) showed strong direct effect of women’s education (OR = 8.14, CI = 3.77–17.57), husband’s education (OR = 2.63, CI = 1.69–4.10) and economic status of household (OR = 1.42, CI = 1.01–2.03). When we adjusted women’s education for husband’s education, the odds ratio decreased around 22% {from (OR = 8.14, CI = 3.77–17.57) to (OR = 6.32, CI = 2.77–14.46)}, and was mediation effect. The economic status of the household also had mediator effect on women’s autonomy through their education (Table 3).

**Table 3:** Influence of women’s education, husband’s education and economic status of household on women’s autonomy.

Variables		Model I*			Model II**			Model III***		
		OR	95% C.I. for OR		OR	95% C.I. for OR		OR	95% C.I. for OR	
			Lower	Upper		Lower	Upper		Lower	Upper
Women’s education status										
	Illiterate/less educated	Ref.			Ref.			Ref.		
	Educated	8.14	3.77	17.57	6.32	2.77	14.46	6.35	2.76	14.58
Husband’s education status										
	Illiterate/less educated	Ref.			Ref.			Ref.		
	Educated				1.50	0.91	2.49	1.51	0.90	2.55
Economic status of household										
	Low economical status							Ref		
	High economical status							0.98	0.67	1.44

* *Model I–Respondent’s education and women’s autonomy*

** *Model II–Respondent’s education and husband education*, *and women’s autonomy*

*** *Model III–Respondent’s education*, *husband’ education and economic status of household*, *and women’s autonomy*

## Discussion

The findings of this study show that the overall women’s autonomy status was low in Kapilvastu district. Decision making autonomy and freedom of movement autonomy scored better than financial autonomy of women. It varied with the demographic and socio-economic characteristics of women. We found significant association of women’s autonomy with higher age difference at marriage, advantaged caste/ethnicity, higher education status of women and their husbands, and better husband’s occupation and economic condition of the family. Women’s education had strong direct association with their autonomy. From the multivariate analysis, we found mediation effect of husband’s education and economic status of the household on the women’s autonomy.

Women’s autonomy correlated with their age, education, occupation, income, single family, closer ties to natal kin in most developing countries [20,21]. A study in India and Pakistan shows that Hindu Indian women had higher autonomy compared to Islamic Pakistani women [21]. Most prior studies focused on women’s age, age at marriage, age difference at marriage, parity, birth order, birth spacing, ethnicity, religion, place of residence and family structure. Some studies included education and occupation status of couple, economic status of the household, women’s property-right, laws which discriminated against women, policies and programs, and political factors [8,9,22].

Considering the subjective nature of women’s autonomy, we included a broad range of demographic and socio-economic variables in the study. Most socio-economic factors were found to be positively associated with their autonomy at the household level. A study in south India shows that education of women, education of husband, age at marriage, marital duration, type of marriage (love marriage), number of children and membership in self-help-group were significantly associated with women’s autonomy at the household level [23]. The findings of the study further show that women who had improved socioeconomic characteristics were more likely to enjoy relatively higher autonomy at the household level.

Woman’s education, husband’s education and economic status of the household were found as key predictors among several demographic and socioeconomic variables. We wanted to assess whether women’s education influences their autonomy independently or interacts with other factors. Constructing multivariate models, we further analyzed various pathways among women’s education, husband’s education and socioeconomic status of the household with women’s autonomy. Woman’s education was found to be a key predictor. Husband’s education and economic status of the household were other important mediating factors of the pathways. It indicates that woman’s education, husband’s education and economic status of the household played a vital role for improving the women’s autonomy in Kapilvastu district.

Despite the population based cross-sectional study, this study reports only quantitative information. Women’s autonomy is a comprehensive subjective phenomenon thus quantitative information may need qualitative explanation for its precise assessment. Similarly, due to constraints of time, and financial and physical resources, we covered minimum required sample size. All findings are based on the reported information of respondents. Considering the limited size of the target population at VDC level, we followed convenience sampling methods for recruiting the respondents at community level. Most readers and researchers often raise a question on convenience sampling method about the representation of population.

## Conclusions

Education status of women is a key predictor of women’s autonomy in Kapilvastu district. Similarly, other important predictors are husband’s education and economic status of the household. Husband’s education and economic status of the household also have mediator effect on women’s autonomy through their education. Improving education status and economic conditions of women and their husbands may be the best solution to promote women’s autonomy.

## Supporting Information

S1 Data File Survey data for developing women’s autonomy measurement scale. (SAV)

S2 Data File Survey data for assessing women’s autonomy status. (SAV)

S1 Table Women’s autonomy measurement scale. (DOCX)
