# Supplementary material for: Women’s Autonomy and Its Correlates in Western Nepal: A Demographic Study
Source: PLoS One. 2016 Jan 22;11(1):e0147473. doi: 10.1371/journal.pone.0147473 (PMC4723155; doi:10.1371/journal.pone.0147473)
Supplement: S1 Table — (DOCX) [file pone.0147473.s003.docx]

| **S1 Table. Women’s Autonomy Measurement Scale**  Select only one out of three given options and write respective number (2 or 1 or 0 as applicable) in the given box. | | | | |
| --- | --- | --- | --- | --- |
| SN | Autonomy domains | Scale | | |
| **A.** | **How do you make decision on-** | **Independent**  **(2)** | **Joint**  **(1)** | **Dependent**  **(0)** |
| 1 | what food should be cooked? |  |  |  |
| 2 | daily household expenditure/purchase? |  |  |  |
| 3 | children’s clothes and food? |  |  |  |
| 4 | children’s education? |  |  |  |
| 5 | children’s and females’ health care and medicine? |  |  |  |
| 6 | inviting and hosting guests? |  |  |  |
| 7 | use of contraceptives? |  |  |  |
| 8 | having baby/another baby? |  |  |  |
| 9 | purchasing major goods in household such as land, house, computer, TV? |  |  |  |
| 10 | being a member of public institutions/organizations? |  |  |  |
| Total score = 0 to 20 | | | | |
| **B.** | **Do you need permission to -** | **Never**  **(2)** | **Sometimes**  **(1)** | **Always**  **(0)** |
| 11 | going outside the house/compound? |  |  |  |
| 12 | going for marketing / shopping? |  |  |  |
| 13 | going to hospital/health care facility? |  |  |  |
| 14 | going to children’s school? |  |  |  |
| 15 | visiting to natal family or relative/s’ house? |  |  |  |
| 16 | visiting friend/s’ house? |  |  |  |
| 17 | going to public places/programmes such as temple, church, other religious places, public programmes? |  |  |  |
| Total score = 0 to 14 | | | | |
| **C.** | **Do you need permission to-** | **Never**  **(2)** | **Sometimes**  **(1)** | **Always**  **(0)** |
| 18 | work outside the house for income |  |  |  |
| 19 | spend money for household affairs |  |  |  |
| 20 | lend/spend money as per personal need and interest |  |  |  |
| 21 | saving money for your future use |  |  |  |
| 22 | handle separate bank account |  |  |  |
| 23 | own and control personal property |  |  |  |
| 24 | give money or goods to natal family |  |  |  |
| Total score = 0 to 14 | | | | |
| Grand total score = 0 to 48 | | | | |
